# Reply to ‘Mercury exposure and Minamata disease in Brazil: evidence or alarm?’

**DOI:** 10.1055/s-0046-1820106

**Published:** 2026-04-28

**Authors:** Gustavo Maximiano-Alves, Eder Leandro da Silva Dantas, Maria Elena Crespo-Lopez, José Luiz Martins do Nascimento

**Affiliations:** 1 Universidade de São Paulo, Faculdade de Medicina de Ribeirão Preto, Departamento de Neurociências e Ciências do Comportamento, Ribeirão Preto SP, Brazil.; 2 Universidade Federal do Pará, Instituto de Ciências Biológicas. Belém PA, Brazil.; 3 Instituto Amazônico do Mercúrio, Belém PA, Brazil.; 4 Instituto Nacional de Ciências e Tecnologia em Neuroimunologia, Rio de Janeiro RJ, Brazil.

Dear Editor,

We would like to thank the authors for their letter of interest in our review 1 and the Editor for the opportunity to comment on it. We welcome scholarly discussion on mercury exposure and its neurological consequences, a topic of growing relevance in Brazil and worldwide.

Our article was conceived as a narrative review and clinical alert, aimed at neurologists, rather than as an epidemiological or outbreak-reporting study. Regarding the latter aspects, the readers can consult systematic and scoping reviews that demonstrate a “widespread outbreak” in the Amazon. 2 3 4 5 Considering that this outbreak has been largely demonstrated, the primary objective of our review was to highlight clinical manifestations and emphasize the persistent problem of underdiagnosis and underreporting, particularly in vulnerable populations in Brazil.

Regarding the title's question, “A Brazilian Minamata disease?”, we stress that it was intentionally posed as a provocative and cautionary inquiry, not as a statement or diagnostic claim. As explicitly discussed in the manuscript, the absence of validated national diagnostic criteria and comprehensive surveillance mechanisms currently precludes the formal characterization of a Minamata-like epidemic in Brazil, despite the large body of scientific evidence already available. 2 3 4 5 Importantly, the lack of official notification is itself a central concern raised by our review. The colleagues' observation that available data points to underreporting and diagnostic gaps aligns with our main argument. In this context, we believe that raising clinical suspicions among neurologists is a necessary first step toward improved case detection and future epidemiological studies.

The adoption of a multidisciplinary approach encompassing environmental, epidemiological, and neurological aspects was intentional and reflects the complex nature of mercury exposure and intoxication. In this context, neurologists cannot disregard the need for multidisciplinary knowledge to adequately treat the patients, although it can be hard at the beginning to learn concepts in environmental and toxicological sciences, as well as to understand the Amazonian reality.

Finally, we appreciate the acknowledgment of the article's relevance and its comprehensive discussion of multiple exposure sources beyond gold mining, including dental amalgams, cosmetics, and dietary intake. We hope that this work contributes to increased awareness, improved diagnostic vigilance, and, ultimately, to more robust research and surveillance efforts aimed at addressing mercury-related neurological diseases in Brazil.
